# Abiraterone acetate in patients with metastatic castration-resistant prostate cancer: long term outcome of the Temporary Authorization for Use programme in France

**DOI:** 10.1186/s12885-015-1257-2

**Published:** 2015-04-04

**Authors:** Nadine Houédé, Philippe Beuzeboc, Sophie Gourgou, Diego Tosi, Laura Moise, Gwenaëlle Gravis, Remy Delva, Aude Fléchon, Igor Latorzeff, Jean-Marc Ferrero, Stéphane Oudard, Sophie Tartas, Brigitte Laguerre, Delphine Topart, Guilhem Roubaud, Hanane Agherbi, Xavier Rebillard, David Azria

**Affiliations:** 1 Department of Medical Oncology, Nîmes University Hospital, Nîmes, France; 2 INSERM U1194, Montpellier, France; 3 Department of Medical Oncology, Curie Institute, Paris, France; 4 Biostatistics Unit, ICM - Montpellier Cancer Institute, Montpellier, France; 5 Department of Medical Oncology, ICM - Montpellier Cancer Institute, Montpellier, France; 6 Department of Medical Oncology, François Baclesse Cancer Centre, Caen, France; 7 Department of Medical Oncology, Paoli Calmette Institute, Marseille, France; 8 Department of Medical Oncology, Paul Papin Cancer Centre, Angers, France; 9 Department of Medical Oncology, Leon Bérard Cancer Centre, Lyon, France; 10 Clinique Pasteur, Toulouse, France; 11 Department of Medical Oncology, Antoine Lacassagne Cancer Centre, Nice, France; 12 Department of Medical Oncology, Georges Pompidou European Hospital, Paris, France; 13 Department of Medical Oncology, Lyon University Hospital, Lyon, France; 14 Department of Medical Oncology, Eugène Marquis Cancer Centre, Rennes, France; 15 Department of Medical Oncology, Montpellier University Hospital, Montpellier, France; 16 Department of Medical Oncology, Bergonié Cancer Institute, Bordeaux, France; 17 Department of Urology, Clinique Beausoleil, Montpellier, France; 18 Department of Radiation Oncology, ICM - Montpellier Cancer Institute, Montpellier, France

**Keywords:** Metastatic castration-resistant prostate cancer, Abiraterone acetate, Efficacy, Prognostic factor

## Abstract

**Background:**

COU-AA-301 trial has proved that abiraterone acetate (AA), a selective inhibitor of androgen biosynthesis, improved overall survival (OS) of patients with metastatic castration resistant prostate cancer (mCRPC) after a first line of docetaxel. Based on this result, a Temporary Authorization for Use (TAU) was performed between December 2010 and July 2011 to provide patients with mCRPC the opportunity to receive AA before its commercialization. The aim of this study was to evaluate safety and efficacy of AA treatment in this TAU.

**Methods:**

Between December 2010 and July 2011, we conducted an ambispective, multicentric cohort study and investigated data from 20 centres participating to the AA TAU for patients presenting mCRPC and already treated by a first line of chemotherapy (CT). Statistical analyses of the data were performed using the Stata software v13 to identify predictive and prognostic factors.

**Results:**

Among the 408 patients, 306 were eligible with a follow-up at 3 years. Median OS was 37.1 months from beginning of CT and 14.6 months from AA introduction. 211 patients (69%) received ≥ 3 months of AA and 95 patients (31%) were treated less than 3 months. In the multivariate analyses, duration of AA was significantly correlated with PSA decrease at 3 months. Additionally, shorter time under AA treatment, presence of multiple sites of metastasis and previous hormonal treatment duration were three independent factors associated with poorer OS. At the time of analysis ten patients were still under treatment for more than 3 years.

**Conclusions:**

Biochemical response monitored by PSA changes at 3 months is a strong predictive factor for AA treatment duration. Some high responders’ patients could beneficiate from AA for more than 3 years.

## Background

Management of metastatic castration-resistant prostate cancer (mCRPC) has dramatically changed over the past 5 years [1]. Until 2011, the standard of care in first line was the addition of docetaxel, a tubulin poison chemotherapy (CT), to LHRH analogue considering that hormonal treatments alone are no longer efficient in this setting [2]. As second line treatment, the only published phase III trial compared the use of cabazitaxel, another tubulin poison, to mitoxantrone after progression and led to the approval of cabazitaxel [3].

New paradigms have emerged in the last decade with initial studies showing that Abiraterone Acetate (AA) may reverse hormonal resistance by specifically inhibiting 17 α-hydroxylase/C17,20 lyase (CYP17A1) involved in the androgen synthesis pathway [4]. Indeed, CYP17A1 is expressed in testicular, adrenal and prostatic tumor tissues, which explain why mCRPC tumor growth still relies on androgen. AA can overcome both “standard” and “backdoor” pathway of androgen synthesis and may result in a drastic decrease of testosterone circulating levels [5]. In that context, the phase III trial COU-AA-301 demonstrated a significant overall survival benefit of AA/prednisone compared to placebo/prednisone (14.8 vs 10.9 months) [6].

In the meantime, France was one of the first countries to make AA available to mCRPC patients after docetaxel CT through a Temporary Authorization for Use (TAU). This type of program allowed patients to have access to the drug from the time of European Medicines Agency approval and the reimbursement approval by the French National Health Services.

In December 2010, we undertook an observational study evaluating safety and long-term efficacy of AA in the daily clinical practice.

## Methods

### Data collection

The French Agency for National Medical Security allowed patients with mCRPC that progressed during or after docetaxel, to access AA before its commercial availability from December 2010 to September 2011.

This ambispective observational cohort study was conducted in 20 centres that accepted to record AA safety and efficacy data for all their patients enrolled in the TAU.

Data collection was done on site from medical records of all the TAU patients. Data were updated in April 2014.

### Ethics statements

This study was approved by the French data protection authorities (CNIL) and the Comité Consultatif sur le Traitement de l’Information en matière de Recherche dans le domaine de la Santé (CCTIRS # 11.545 approved on September 29th 2011). Written informed consent was waived because this is a retrospective study. The study was undertaken in accordance with the ethical standards of the World Medical Association Declaration of Helsinki.

### Patients and treatment

Inclusion criteria were as follows: men with mCRPC and documented disease progression during or after a docetaxel-containing regimen. Progression was defined by clinical progression, PSA progression and/or radiographic progression on bone scan or CT scan, as defined by the Prostate cancer Working Group 2 (PCWG2) criteria [7]. Patients should be under androgen deprivation and had castration level of testosterone (<50 ng/ml). Before AA delivering, patients should had potassium level >3.5 mmol/l, ASAT/ALAT <5 UNL in case of liver metastasis, or <2.5 UNL in the absence of metastases, and total bilirubin <1.5 UNL. Regarding the toxicity profile, exclusion criteria included uncontrolled hypertension, severe or unstable angina, and myocardial infarction within 6 months, heart failure, arterial or venous thromboembolic events, or clinically significant ventricular arrhythmias.

The recommended dose of AA was 1 g per day, as 4 tablets of 250 mg in one administration one hour before or two hours after a meal, in combination with oral prednisone 5 mg twice a day. Patients were treated until clinical, biological or radiological progression according to PCWG2 criteria, death, unacceptable toxicity, or physician’s or patient’s decision to stop the treatment.

### Outcomes measures

In the context of this TAU, clinical and biological follow-up were scheduled every 15 days within the first three months of treatment and monthly afterwards until treatment discontinuation. Radiological evaluation during follow-up was not mandatory. All selected variables were collected in the medical report i.e. patients’ characteristics, disease description at diagnosis (Gleason score, tumour classification, metastasis sites before chemotherapy and before AA, PSA kinetics, number of prior docetaxel cycles, duration of treatments and reasons for treatment discontinuation), and follow-up.

For the efficacy analysis, survival time were calculated in two different manners: from the beginning of CT, defined as the time interval between the start of first line chemotherapy and the date of death; and from the initiation of AA and the date of death. Patients alive were censored at the last known follow-up date. AA treatment duration was classified in three categories (≤3 month, 3–6 months, and > 6 months), according to the biological and radiological assessment planned in the TAU program, and in two categories (≤3 month, >3 months) for multivariate analysis. PSA was measured at the time of inclusion, at 3 and 6 months as suggested in the TAU. Adverse events were followed on a monthly basis and graded according to the NCI-CTCAE v3.0.

### Statistical methods

Qualitative variables were described by frequency of modalities and percentage. Continuous variables were described by mean, median, and range. Data are presented with 95% confidence intervals (95% CI), calculated with the use of exact methods based on the binomial distribution for discrete variables.

Median follow-up was calculated with the use of the Kaplan-Meier reverse method.

Predictive factors of AA treatment duration (in two categories: ≤3 month, >3 months) were identified with the use of univariate and multivariate logistic regression using a backward selection method, including the following variables: age, Gleason score, duration of CT before AA treatment, baseline PSA, PSA before AA treatment, site of metastasis before AA treatment initiation, duration of hormone therapies, and number of CT lines.

Overall survival rates were estimated using the Kaplan–Meier method.

A Cox proportional-hazards model was used to estimate the hazard ratios indicating the effects of prognostic factors on the risk of death. Three and 6 months landmark analyses were performed to explore the association between duration of AA treatment and overall survival.

All tests were two-sided, with a P value of less than 0.05 considered as statistically significant. Analyses were performed using the Stata software, v13.

## Results

### Patients

Up to September 2011, 408 patients were enrolled in the initial study. Complete follow-up data were obtained for 306 patients in 13 centres from the 20 initially selected centres and were considered for this report. Seven centres did not want to pursue this observational study.

Median follow-up from the initiation of AA is 36.3 months (95%CI 35.8-37.1). Descriptive data at the time of AA introduction are included in Table 1. Patients’ characteristics were collected at inclusion. Median (range) age was 63 years (46–82). Before starting AA, 41.5% of the patients had bone metastasis only, 9.8% visceral metastasis only, and 48.7% showed multiple sites. Median duration of hormone therapy before chemotherapy was 31.6 months [0–201]. Before starting AA, all patients received at least one line of CT. For most of them, CT was based on docetaxel alone or in combination (298 patients, 97.4%). One hundred seventy (55.6%) patients received only one previous line of CT, 103 (33.7%) two lines, 20 (6.5%) three lines, 10 (3.3%) four lines, and three patients (1%) received five lines. For the patients receiving only one line, median duration of CT was 4.9 months [0–24]. Hundred sixty nine patients (55%) received at least one line of CT post AA treatment (Table 1).

**Table 1:** **Patients’ characteristics and pre-AA history**

Population (N = 306)		
		**Median [range]**
**Age**		63 [46–82]
	**N**	(%)
**Initial Gleason score**		
4 - 6	33	(10.8%)
7	92	(30.0%)
8 - 10	134	(43.8%)
missing	47	(15.4%)
**Sites of metastasis before CT**		
Bone only	144	(47.1%)
Visceral only	57	(18.6%)
Multiple	105	(34.3%)
Bone	246	(80.4%)
Nodes	133	(43.5%)
Lung	22	(7.2%)
Liver	17	(5.6%)
Brain	1	(0.3%)
Other	13	(4.3%)
**Sites of metastasis before AA**		
Bone only	127	(41.5%)
Visceral only	30	(9.8%)
Multiple	149	(48.7%)
Bone	275	(89.9%)
Nodes	146	(47.7%)
Lung	34	(11.1%)
Liver	26	(8.5%)
Brain	5	(1.6%)
Other	18	(5.9%)
**PSA before CT (ng/mL)**	**Median [range]**	**Missing**
	45.4 [0–4967]	37 (12.1%)
**PSA before AA (ng/mL)**	**Median [range]**	
	121.2 [0.15 - 8322]	13 (4.2%)
**Hormone treatment duration (months)**	**Median [range]**	
	31.6 [0–201]	
**CT treatment duration if one line (months)**	**Median [range**	
	4.9 [0.3-20.7]	
**CT treatment duration if more than 1 line (months)**	**Median [95% CI]**	
	6.2 [0 – 50.2]	
**Lines of CT before AA**	**Median [range]**	
	1 [1-5]	
**Number of lines of CT before AA**	**N**	**%**
1	170	55.6
2	103	33.7
3	20	6.5
4	10	3.3
5	3	1.0
**Number of lines of CT after AA**	**N**	**%**
0	139	45.4
1	79	25.8
2	52	17
3	29	9.5
4	7	2.3
**First line CT after AA (if applicable)**	**N**	**%**
Cabazitaxel	51	30.6
Docetaxel rechallenge	44	26.4
Distilbene	17	10.2
Mitoxantrone	14	8.5
Enzalutamide	13	7.7
Other	28	16.6

### Efficacy

#### Treatment duration

Median duration of AA treatment was 5.2 months (0.03-34.1).

A total of 211 (69%) patients received more than 3 months of AA and 10 patients were still under treatment at the time of the last follow-up visit (April 2014) with a median (range) duration of 36.5 months (32.9-38.9) (Table 2).

**Table 2:** **Treatment duration of Abiraterone Acetate**

*Months*	*N*	*%*
≤ 3	85	27.8
]3-6]	84	27.4
>6	127	41.5
Ongoing treatment	10	3.3

#### Overall survival

**Figure 1:**
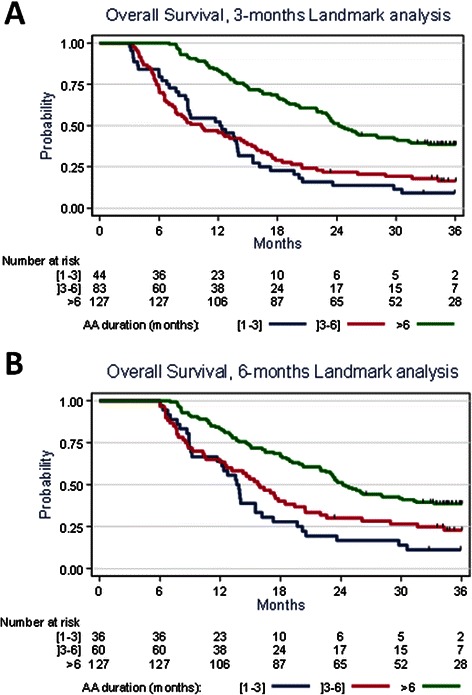
**Overall survival from the beginning of Abiraterone Acetate for the three categories of patients (treatment duration ≤3 months, [3,6], >6 months). (A)** 3 months Landmark analysis. **(B)** 6 months Landmark analysis.

OS from the beginning of CT and from the initiation of AA were 37.1 months (95% CI 32.5- 39.7) and 14.6 months (95% CI 12.6- 16.5), respectively. OS was significantly associated with the duration of AA (*P* < 0.001) in both the 3 months and 6 months Landmark analyses (Figure 1A & B).

#### Biological response

**Figure 2:**
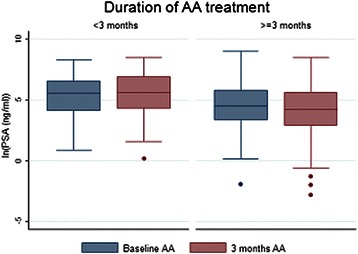
**Changes of PSA values between baseline (blue boxes) and 3-months (red boxes) for patients receiving <3 months (left panel) or > = 3 months (right panel) of AA.** The dots correspond to extreme values of PSA levels.

In the overall population, median PSA value at baseline was 121.2 ng/ml [0.15-8322], 87.8 ng/ml [0–5001] at month 3, and 79 ng/ml[0–5600] at month 6. A subgroup analysis was performed to assess PSA changes between baseline and month 3 for patients receiving <3 months (97 patients) and > = 3 months (211 patients) of AA treatment (Figure 2). The results show that the PSA response for patients who were treated more than three months by AA was significantly higher (*P* = 0.00025) than for patients who were treated less than three months (Figure 2).

One hundred eighty five patients (60.4%) received one to three treatments following AA: cabazitaxel for 64 patients (21.7%), rechallenge docetaxel (n = 60, 20.3%), enzalutamide (n = 31, 10.5%), cyclophosphamide (n = 27, 9%), mitoxantrone (n = 24, 8.1%), and estramustine (n = 13, 4.4%).

At the time of the last follow-up visit (April 2014) 10 patients, treated in 4 different centres, were still under AA. For this long-term responder subpopulation, median age was 65 years [54–78]; Gleason score at the beginning of AA was 6 for two patients, 8 for five patients and 9 for one patient, missing data for 2 patients. All of them had bone metastases but four presented concomitant visceral metastases. Median PSA value was 33 ng/ml [0.15-231] at baseline, 3.4 ng/ml [0.14-170] at month 3, and 1.34 ng/ml [0.15-231] at month 6.

#### Safety

Most common adverse events were hypokalaemia (n = 16 but grade ≥3 for 2 patients), hypertension (n = 9 but grade ≥3 for 1 patient), hepatic and liver dysfunction (n = 6 but grade ≥3 for 2 patients). Treatment was safely administered with only seventeen adverse events resulting in treatment discontinuation. Treatment was discontinued for 274 (89.5%) patients because of disease progression. Among them, 26 patients (9%) died from their disease and three patients (1%) died from another cause.

### Predictive and prognostic factors

#### Landmark analyses included 264 patients followed for more than 3 months and 233 patients followed for more than 6 months.

In univariate analysis, predictor of duration of AA treatment was PSA changes between the start of AA and the 3 months time point (*P* < 0.0001). The multivariate analysis confirmed a longer AA treatment in case of PSA decrease under treatment (OR 0.13, *P <* 0.0001) (Table 3).

**Table 3:** **Predictive factors of AA treatment duration**

		Univariate analysis		Multivariate analysis	
		OR	p	OR	p
Age	<60 vs	1	0.107		
	> = 60	0.57 95% CI [0.29-1.13]			
Gleason score	4-6	1			
	7	1.24 95% CI [0.45-3.39]	0.68		
	8-10	1.16 95% CI [0.44-3.05]	0.76		
Duration of CT before AA treatment	<=4 months	1			
	]4-6]	0.99 95% [0.42-2.31]	0.98		
	[6-9]	2.00 95% [0.81-4.98]	0.13		
	>9	1.17 95% [0.53-2.59]	0.70		
PSA baseline before CT	Continuous variable	0.99 95% [0.99-1.00]	0.10		
PSA baseline before AA	Continuous variable	1.00 95% [0.99-1.00]	0.67		
Sites of metastasis	Bone or visceral alone vs Multiple	1	0.17	1	
		0.65 95% CI [0.36-1.19]		0.71 95% [0.35-1.44]	0.34
Number of CT lines	1	1			
	2	1.02 95% [0.53-1.99]	0.94		
	3	1.23 95% [0.33-4.56]	0.75		
	4-5	0.26 95% [0.02-4.34]	0.35		
PSA variation at 3 months	Decrease	1	<0.0001	1	
	Increase	0.06 95% [0.02-0.19]		0.13 95% [0.06-0.31]	<0.0001

Three factors were found to be associated with poorer OS following univariate analysis: multiple sites of metastasis (versus bone metastasis alone) (*P* =0.025), previous hormonal treatment duration (less than 70 months; 75th percentile) (*P* =0.001) and duration of AA treatment (less than 3 months) (*P* < 0.001). Similar results were obtained in the multivariate analysis with the following significant associations: multiple sites of metastases (*P* =0.019, HR 1.41 [95% CI 1.05-1.88]), first line hormonal treatment duration (*P* =0.001, HR 0.54 [95% CI 0.38-0.77]) and duration of AA treatment (*P* <0.001, HR 0.55 [95% CI 0.39-0.77]) (Table 4).

**Table 4:** **Pronostic factors of overall survival (Cox model)**

		Univariate analysis		Multivariate analysis	
		HR	p	HR	p
Gleason score	4-6	1			
	7	1.54 95% CI [0.91-2.60]	0.11		
	8-10	1.63 95% CI [0.98-2.72]	0.06		
Nb CT line	1	1			
	2	1.17 95% CI [0.86-1.60]	0.309		
	3	1.09 95% CI [0.59-2.03]	0.785		
	4	1.11 95% CI [0.52-2.39]	0.788		
	5	0.81 95% CI [0.11-5.82]	0.835		
Sites of metastasis	Bone or visceral alone vs Multiple	1	0.025	1	0.019
		1.38		1.41	
		95% CI [1.04-1.83]		95% CI [1.05-1.88]	
Previous hormonal treatment duration	Less than 70 months vs More than 70 months	1	0.001	1	0.001
		0.55		0.54	
		95% CI [0.39-0.79]		95% CI [0.38-0.77]	
Duration of AA treatment	Less than 3 months vs More than 3 months	1	<0.001	1	<0.001
		0.52		0.55	
		95% CI [0.38-0.74]		95% CI [0.39-0.77]	

## Discussion

This ambispective observational cohort study enrolled all the eligible mCRPC patients of the 20 centres which agreed to participate. This was rapidly followed by the prescription of AA by other centres leading to a national TAU cohort of a total of 1629 patients over nine months. When the TAU was initiated, no other treatment was available besides docetaxel or experimental treatments accessible in clinical trials. Therefore, a high number of patients were allowed to receive AA treatment. Consequently, the population of this study is a “real-life”, non-selective population that includes a large number of patients with advanced disease (48.7% with multiple sites of metastases) who received up to 5 lines of chemotherapy.

In terms of safety, the pivotal COU-AA-301 study demonstrated that AA was associated with elevated mineral corticoids levels, aminotransferase level affecting liver function, urinary tract infections, fluid retentions, and oedema [6]. In our study, a high proportion of included patients presented an advanced disease, but no new adverse event was recorded, confirming the safety of AA usage.

Median treatment duration was three months shorter than the one observed in the COU-AA-301 trial (5 versus 8 months). Though patients were more heavily pre-treated and the duration of treatment by AA was much shorter, we did not observe a significant change in OS (14.6 months in the present study versus 14.8 months in the COU-AA-301). In concordance with the OS that is observed from the introduction of first line CT, it may reflect the evolution of care in the management of mCRPC patients.

We found that the duration of AA treatment was significantly associated with prolonged survival. Two third of the patients received more than 3 months of AA, whereas the other third received less than 3 months of AA, indicating that these patients rapidly developed a resistance to the drug. This resistance is mainly due to an alteration of the androgen receptor (AR) axis by several mechanisms including changes in AR expression levels, occurrence of AR mutations, interactions of AR with co-activators or co-repressors, or increase in the expression of the CYP17A1 target itself [8]. In these patients, a fatal issue is rapidly observed despite the use of cabazitaxel or of the AR antagonist enzalutamide. Indeed, several retrospective studies showed that enzalutamide had modest clinical activity in patients with mCRPC who previously received docetaxel and AA [9,10]. For patients where resistance is due to an overexpression of CYP17A1, it is however possible to envisage an increase in AA dosage in order to prolong survival [11].

Prior to our study, the only relevant predictive factor of response to AA was the baseline level of testosterone as determined in the post hoc exploratory analysis of COU-AA-301 data, the OS being significantly longer in patients with high androgen levels [12,13]. Interestingly, we found that the main predictive factor of AA benefit was the difference in PSA values between baseline and 3 months of treatment. PSA flare up described previously concerns a minority of patients (less than 10%) [14], so determination of PSA levels could help the early monitoring of AA benefit and avoid maintaining an ineffective costly treatment. When localized to the bone only, presence of metastases was a good prognostic factor significantly associated with prolonged OS. A former retrospective analysis with 116 patients treated with AA at the Princess Margaret Hospital in Toronto showed that bone localisation could impact PSA response [15] further strengthening our current observation. We also report for the first time that duration of hormonal sensitivity was associated with prolonged survival following AA treatment, asking the question whether AA should be prescribed for patients with particularly high levels of resistance to hormonal therapy.

Recent studies have shown new options for the treatment of mCRPC: the use of AA as first line treatment in chemo-naïve patients [13] or the use of enzalutamide as first [16] or second line [17] treatment. However, there is no study evaluating the different possible sequences with the three drugs that are currently approved or going to be approved as first line treatment. Preclinical data showed impaired efficacy of docetaxel and cabazitaxel in abiraterone-resistant prostate cancer cell lines [18]. These data were reinforced by clinical studies evidencing a lower activity of docetaxel in patients pre-treated with AA [19,20]. Thus, the question of AA positioning in terms of clinical benefit in a chronic disease where patients could live up to 3 years remains open. The results of our study tend to suggest that using AA post docetaxel is an excellent option with a median OS of 37 months.

## Conclusions

Our study provides new information for current clinical practice by showing that patients with progressive disease within the first 3 months of AA treatment will probably present short overall survival. It further shows the utility of a strong monitoring of the PSA changes that could act as an early predictive marker of this clinical benefit and may encourage physicians to switch rapidly to other therapies. Results of other on-going observational studies are awaited to confirm which patients could beneficiate the most from AA [21].

## Abbreviations

AA: Abiraterone Acetate
ALAT: Alanine Aminotransferase
AR: Androgen Receptor
ASAT: Aspartate Aminotransferase
CT: Chemotherapy
CYP17A1: Cytochrome P450, Family 17, Subfamily A, Polypeptide 1 (17 α-hydroxylase/C17,20 lyase)
LHRH: Luteinizing Hormone Releasing Hormone
mCRPC: metastatic Castration-Resistant Prostate Cancer
NCI-CTCAE: National Cancer Institute-Common Terminology Criteria for Adverse Events
OS: Overall Survival
PCWG2: Prostate cancer Working Group 2
PSA: Prostate Specific Antigen
TAU: Temporary Authorization for Use
